# The Serotonergic System: Its Role in Pathogenesis and Early Developmental Treatment of Autism

**DOI:** 10.2174/157015909788848848

**Published:** 2009-06

**Authors:** D.I Zafeiriou, A Ververi, E Vargiami

**Affiliations:** 1st Department of Pediatrics, Aristotle University of Thessaloniki, Greece

**Keywords:** Autism, serotonin, serotonergic, developmental intervention, plasticity, 5-hydroxytryptophan.

## Abstract

Autism is a severe childhood disorder already presenting in the first 3 years of life and, therefore, strongly correlated with neurodevelopmental alterations in prenatal, as well as postnatal period. Neurotransmitters hold a pivotal role in development by providing the stimulation needed for synapses and neuronal networks to be formed during the critical period of neuroplasticity. Aberrations of the serotonergic system modify key processes in the developing brain and are strongly implicated in the pathophysiology of developmental disorders. Evidence for the role of serotonin in autism emerges from neuropathological, imaging and genetic studies. Due to its developmental arrest, autism requires early intervention that would, among others, target the disrupted serotonergic system and utilize brain plasticity to elicit clinically important brain changes in children.

## INTRODUCTION

Autism is a severe childhood disorder presenting in the first 3 years of life and affecting 1.3/1000 individuals [41]. It is the most common of pervasive developmental disorders (PDDs), which also include Rett disorder, Asperger’s syndrome, childhood disintegrative disorder and pervasive developmental disorder not otherwise specified [1,106]. Autism shows a high gender repartition with a male/female ratio varying from 1.33 to 16.0 in different studies and a mean male/female ratio of 4.3:1 [42], whereas it is characterized by deficit in three main areas: social interaction, verbal and non-verbal communication and restriction of activities and interests repertoire [105]. The symptoms spectrum is rather wide, including unusual sensory responses and motor patterns, pain insensitivity, gastrointestinal dysfunction, anxiety, depression, sleeping disturbances, attention issues and aggression or self-injury [69,106].

A variety of causal factors have been related to autism, including heredity, perinatal and environmental incidences, neuroanatomical changes, neurotransmitter aberrations etc. According to growing evidence, autism is a disorder of developmental arrest, rather than a progressive process and requires, therefore, the earliest possible intervention.

Neurotransmitter function in autism has long been under intense research, given the pivotal role of neurotransmitters in brain maturation and cortical organization [77]. The disruption of the serotonergic system is one of the most consistent and well-replicated findings in autism.

Serotonin is synthesized from the essential aminoacid tryptophan. L-tryptophan is hydroxylated to 5-hydroxytryptophan (5-HTP) (by the rate-limiting enzyme tryptophan hydroxylase), which is subsequently decarboxylated by aromatic-L-amino acid decarboxylase to be, eventually, converted to serotonin. Serotonin is cleared by the lung and the liver, except for a small amount that remains circulating and is stored in platelets. Serotonin does not cross the blood-brain barrier and, therefore, the brain serotonin is exclusively synthesized in the central nervous system (CNS). It is catabolized there by monoamine oxidase A and the product is secreted into blood [70].

The serotonergic system is one of the most widely distributed, as well as one of the earliest to develop in the mammalian embryo. The majority of neurons are located in the median and dorsal raphe nuclei. The first mainly provides fibres to the cortex and the latter to the hippocampus. The serotonergic system innervates almost all areas of the brain, whereas serotonin presents in non-serotonergic cells, as well, where it acts as a developmental signal [89]. Serotonergic neurons can be detected in the human brain since the 5^th^ gestational week [92] and during the following months they grow and multiply rapidly.

The early appearance of the serotonergic system, as well as its intense activity during the first stages of development, indicate its role in the developmental process. Serotonin is reported to influence neurogenesis and/or neuronal removal, neuronal differentiation, synaptogenesis etc [103]. Among others, the serotonergic system modulates the activity of GABAergic interneurons, particularly the Cajal-Retzius cells, whereas it is released by axons projecting from the thalamus, thus playing a critical role in the establishment of thalamocortical afferents [10]. In addition, serotonin holds an important role in dendritic development, including overall dendritic length, spine formation and branching in both hippocampus and cortex [103]. As far as it concerns the cortex, the serotonergic system also regulates the development of the barrel fields, which constitute an area of the primary somatosensory cortex and demonstrate a transient expression of serotonin terminals [103]. The early disturbance of the serotonergic system disrupts the developmental process and contributes to several of the neuropathological changes in autism, as discussed below.

Serotonin has at least 14 different receptors, almost all of which are also involved in development. Activation of 5HT_1A_ receptors influences the length of dendrites and the number of dendritic spines in hippocampus [87], whereas the 5HT_2C_ receptors are involved in long-term potentiation in the hippocampus [94] and the 5HT_2A_ in neuronal differentiation, dendritic maturation and modulation of brain-derived neurotrophic factor levels [39,96].

Apart from its role in development, serotonin is a signalling molecule with a widespread distribution in the CNS, thus influencing almost every sphere of the mammalian physiology: cardiovascular regulation, respiration, gastrointestinal system, pain sensitivity, thermoregulation, circadian rhythm, appetite, aggression, sensorimotor activity, sexual behavior,mood, cognition, learning and memory [89]. Therefore, several symptoms caused by a possible serotonergic disturbance would be common with the most usual characteristics of autism.

Increase of platelet serotonin was the first finding to imply the involvement of the serotonergic system in the pathogenesis of autism. Literature suggests that hyperserotonemia is present in 25-50% of individuals with autism, as well as their first-degree relatives [2,27], whereas parents of children with autism are more likely to present serotonin-related psychiatric disorders, such as depression or obsessive compulsive disorder [27,34].

Nevertheless the elevation in blood, there are indications that serotonin is actually decreased in the brain. Tryptophan depletion experiments have led to exacerbation of many repetitive behaviors (particularly whirling, flapping, pacing, banging and hitting themselves, rocking and anxiety) [64], whereas serotonergic agents, such as selective serotonin reuptake inhibitors (SSRIs), have proven beneficial for patients with autism [57]. The reported decrease in the ratio of serum tryptophan to large neutral amino-acids probably indicates a low availability of tryptophan in the autistic brain [33], whereas administration of 5-HTP has led to substantial increase of serotonin concentration in blood of patients, contrary to controls, on whom it had no impact [31]. In addition, animal studies have identified a serotonin depletion model of neonatal mice that mimics neurochemical and structural changes in cortex and also displays a behavioral phenotype consistent with autism [14,47]. As far as it concerns humans, it has been reported that the severity of at least one specific behavioral dimension in autism (repetitive behaviors) parallels the sensitivity of the 5HT_1D_ receptor [48].

Neuroimaging, neuropathological and genetic studies have added to the evidence for the large-scale involvement of serotonin in pathogenesis and, therefore, also in treatment of autism.

## NEUROIMAGING STUDIES

Functional neuroimaging studies have proven the decrease of serotonin synthesis in children with autism aged 2-5 years, as well as the focal aberrations of serotonin synthesis and the low binding potentials of the serotonin transporter and receptor in individuals with PDDs.

Functional neuroimaging studies have shed light into synthesis, as well as binding capacity, of serotonin in individuals with autism. According to positron emission tomography (PET) studies, children aged 2 to 5 years normally undergo a period of increased serotonin synthesis capacity (200% of adult values), followed by a decline toward adult values between the ages of 5 and 14 years. In contrast, patients with autism demonstrate -until the age of 5- a reduced capacity for synthesis, which, however, increases to reach and overcome adults’ values by the age of 15 [23]. Therefore, the hyperserotonemia, which probably appears in older patients, does not actually develop before an age when most diagnoses of autism have been already made [45].

It has been indicated that the developmental pattern of serotonin synthesis in non-autistic children strongly resembles the profile of synaptic density in their frontal cortex, as demonstrated in PET and post mortem studies respectively [10]. The normal process of high brain serotonin synthesis and synaptogenesis during preschool years is highly disrupted in children with autism.

Apart from global abnormalities, PET studies have also revealed focal aberrations of serotonin synthesis with cortical asymmetry in serotonin uptake. Patients with reduced synthesis on the left side demonstrate high frequency of severe language impairment, whereas the decrease on the right side represents higher frequency of left- or mixed-handedness. The asymmetries have been attributed, among others, to the general developmental misregulation of serotonin synthesis or to an early damage of the left dominant hemisphere, leading to compensatory changes on the right side [20].

In addition, studies with single-photon emission computed tomography (SPECT) have revealed the low binding capacity of the serotonin transporter in the medial frontal cortex, midbrain, and temporal lobe areas [63], whereas PET and SPECT investigations have determined the reduced binding capacity of the cortical serotonin type 2-receptor in individuals with PDDs, as well as parents of children with autism [43,71]. The low binding potentials have been attributed to the diminished serotonergic nerve terminals and sparse synapse density.

## NEUROPATHOLOGICAL STUDIES

The serotonergic disruption during development is involved in some of the most consistent neuropathological findings in the autistic brain, such as the alteration of the minicolumns, the limbic system and the cerebellum.

One of the major neuropathological findings in the autistic brain is the disruption of neocortical minicolumns, which are the primary anatomical units with the lowest level of neocortical modularity. The disruption of minicolumns leads to aberrant cortical organization, clinically correlated with altered information processing and perhaps with the “hyperspecific” autistic brain. Minicolumns in autistic brains have been reported to be more numerous, but smaller and closely spaced, compared to controls.  Furthermore, their constituent neurons are more dispersed, accounting for a normal cellular density, whereas the surrounding neuropil is significantly reduced [17,18]. The very detailed focusing, along with the failure to recognize broader contexts of information are probably some of the functional consequences of narrow minicolumns [10]. A tentative case for the explanation of this narrowing can be made for serotonergic disturbances [45].

Serotonin has been recently shown to hold a pivotal role in corticogenesis, mainly through the serotonergic innervation of Cajal Retzius cells in the cortex. Cajal Retzius are reelin-expressing neurons modulating the cortical laminar and columnar organization [74]. Reelin has been long shown to be reduced in autism [40]. Rodent studies with early manipulation of the serotonergic system, causing or mimicking a serotonin increase, have reported disruption of the serotonergic input to Cajal Retzius cells, reduction of brain reelin levels [53] and perturbing of cortical organization [53,107]. Conversely, depletion of serotonin in neonatal rodents has caused delayed development [76] and aberrant appearance of the thalamocortical pattern [12], as well as decreased size of the barrel fields [73,79]. It is noteworthy that during the first postnatal days, rodents show a peak in serotonin levels, similar to the increased serotonin synthesis of humans at the age of 2 years [77].

Another well replicated finding in autism is the alteration of the amygdala, affecting its volume, cellpacking density and activation during processing [46,90]. The central nucleus of the amygdala receives intense innervation from serotonergic dorsal raphe neurons. Rodent studies with prenatal and neonatal manipulation of serotonin (mimicking hyperserotonemia) have reported an increase in calcitonin-gene related peptide (CGRP) in the central nucleus of the amygdala [104]. CGRP is probably involved in conditioned responses to acoustic or somatosensory stimuli and may play a role in fear conditioning [56]. The amygdala is considered to hold a significant role in autism, mainly through its involvement in the developmental deficit in emotion processing and, generally, in social perception [85].

Animal studies with neonatal serotonin depletion have shown a significant decrease of synaptic spine density in the hippocampus [108], as well as a reduction in serotonergic innervation to the hippocampus and the cortex [47]. The hippocampus is critical, among others, for episodic memory, which has been repeatedly reported to be impaired in autism [59]. It has been, additionally, shown that the autistic hippocampus has reduced neuronal cell size and increased cell-packing density [7].

Rodent studies with prenatal and neonatal manipulation of serotonin (mimicking hyperserotonemia) have also proven a loss of oxytocin-containing cells in the hypothalamus [104]. Among other actions, oxytocin is involved in social recognition and affiliative (or prosocial) behaviors [22], which are severely disturbed in autism.

As far as it concerns the cerebellum, its transiently high expression of serotonin receptors in rodents during the first 2 postnatal weeks suggests an association between the decrease in Purkinje cells and the altered serotonergic system [5,24,68]. In addition, it has been reported that mice lacking the gene for reelin and, consequently, the normal reelin levels, demonstrate a reduction in Purkinje cells [10]. Brain reelin levels are affected not only by the lack of the reelin gene, but also by the serotonergic disruption [53], which may, therefore, be involved in the decrease of Purkinje neurons. The reduction of Purkinje cells is one of the most apparent and consistent neuropathologic findings in autism [7].

## GENETIC STUDIES

Molecular approaches with case-control and family-based studies have revealed a number of serotonin-related candidate genes in autism.  Some of them are, additionally, involved in the appearance of gender differences in PDDs.

Tryptophan hydroxylase-2 (TPH2) is the rate limiting enzyme for CNS serotonin synthesis and is encoded by *TPH2*, whereas *TPH1* encodes the peripheral isoform of the enzyme. Only one report has provided preliminary evidence for the involvement of *TPH2* variants in autism [29], whereas following research has failed to confirm the association for either *TPH1* or *TPH2* [81,83]. Nevertheless, both genes have been shown to hold critical roles in the homeostasis of peripheral and brain serotonin respectively [8,32,51].

*SLC6A4* is a second candidate gene with a repeatedly reported causal link to autism [21,28,38,80,93,98,101]. It is located on 17q.11 and encodes the serotonin transporter. It contains over 20 polymorphisms, of which two are the most interesting in neuropsychiatry: 5-HTTLPR and VNTR [72]. 5-HTTLPR is a promoter region polymorphism reported to influence cerebral cortical grey matter volume [98], hippocampal volume and amygdala response [86]. In addition, it has been associated with the presence of conduct disorder, aggressivity and ADHD only in male individuals [15]. However, recent genetic studies, as well as a meta-analysis on polymorphisms’ relation to autism, have produced controversial results, raising concerns regarding the involvement of *SLC6A4* in autism [21,52,82]. The inconsistent findings could be associated with ethnic diversity, methods of genetic analysis and genetic heterogeneity of the disorder [21]. It is noteworthy that the serotonin transporter is expressed in the placenta [6] and, therefore, a maternal modifier effect could potentially pose risk for autism in the child [20].

Evidence for an association with autism exists for several serotonin receptors genes: *HTR1B* [75], *HTR2A* [21], *HTR3A* [3], *HTR5A* [30] and *HTR7* [32], whereas other studies have reported controversial findings [97]. Ongoing and future investigations will probably replicate or produce new results.

*MAOA*, the gene encoding monoamine oxidase, contains a promoter polymorphism (upstream variable number tandem repeats, uVNTR) with a high and a low activity allele. The latter has been consistently associated with cortical enlargement [36], as well as lower intelligence and more severe autistic behaviour [25] in children with autism, whereas, in healthy individuals, it has been related to increased activity in the amygdala and hippocampus and impaired cingulate activation during cognitive inhibition [66]. The *MAOA* polymorphism has been also reported to have a modifying effect in the intelligence of children with autism through the intrauterine environment [54]. In addition, an association of autism with several *MAOA* haplotypes was recently identified, thus suggesting the presence of a causable polymorphism in the haplotype block [109].

The b3-integrin gene *ITGB3* is identified as a quantitative trait locus for blood serotonin levels, primarily in males. *ITGB3* has been reported to show genetic and expression interaction with *SLC6A4*, leading to increase of autism susceptibility [30,101]. Other interactions involving *SLC6A4* with *TPH1* or *HTR1D* or *HTR5A*, as well as *ITGB3* with *HTR1A* or *HTR5A* and *TPH1* with *MAOA*, have been also identified [30, 51].

It should be noted that genetic studies of the serotonergic system have revealed a number of gender-related differences. 5-HTTLRP is associated with the presence of aggressiveness and ADHD in males only [15] and *MAOA* is located on the X chromosome [99], whereas the Leu33 allele in *ITGB3* has been suggested to have a dominant effect in males and a recessive effect in females, as far as it concerns association to autism [100]. In addition, human and animal studies have respectively shown that *ITGB3* [55,84], as well as *SLC6A4* [78], mRNA expressions are reduced by estrogens. Biochemical and neuroendocrine studies have also demonstrated sex related differences. For example, the mean whole blood serotonin level is higher in women [99], the female serotonergic system shows greater response to stimulation with serotonergic agents [88] and SSRIs are more effective in treating women with depression [58]. As far as it concerns development, sex-differences of the serotonergic system have been reported to present rather early and to contribute to dimorphic cortical development [26]. It is, therefore, possible that the serotonergic disturbances are involved in the appearance of the gender differences in autism.

## NEURODEVELOPMENTAL INTERVENTION

Early developmental intervention is of ultimate importance in order to utilize brain plasticity and provide permanent and substantial benefit. However, the administration of any pharmacological treatment to children would first require a large number of animal studies, in order to assess the balance between possible gain and damage.

Another line of research on the serotonergic involvement in autism comes from pharmacological intervention with serotonergic agents.

A significant loss of serotonin terminals, along with decreased metabolic activity in cortex, seizures and “autistic-like” behaviors have been noted in rats, which had been administered with a serotonin agonist, 5-methoxytryptamine, during early development. The rat hyperserotonemia model is consistent with autistic humans during the very early stages of development, when the still incomplete blood-brain barrier allows high blood serotonin to enter the brain and cause the loss of serotonin terminals through a negative feedback effect [65].

Regardless the underlying mechanisms of the serotonin disruption, it has been repeatedly reported that SSRIs demonstrate therapeutic benefit in patients with PDDs. SSRIs influence both peripheral and central levels of serotonin [57] and have been utilized against various symptoms of the disorder [37,49,50]. Although they do not directly affect communication and social deficits, they do improve repetitive behaviours [50] and behavioural control, thus resulting in “prosocial behaviour and subsequent progress in communication” [57]. A number of symptoms (aggression, self-injury, stereotypies, irritability, temper tantrums) are reported to improve, whereas adverse effects include aggressiveness, agitation, motor hyperactivity, sedation, insomnia, anxiety, headaches, appetite changes, behavioral activation etc [57].

Intense controversy exists regarding the use of SSRIs in children. Due to its neurodevelopmental arrest, autism requires rather early intervention that would elicit clinically important brain changes in children. Reports on SSRIs adverse effects, especially when the exposure occurs in utero or in early childhood are controversial [9,10], whereas rodent studies have reported potential late-emerging adverse effects after early administration of SSRIs [4]. Increased extracellular serotonin levels during the perinatal period of mice are associated with subtle changes in brain circuitry and maladaptive behaviours, which are maintained into adulthood [13]. Up to now few data exist on SSRIs use in preschool age or even childhood [57]. In addition, the Food and Drug Administration has issued a controversial public health warning about a causal link between the use of SSRIs and paediatric suicidality [44].

A number of clinical studies would be necessary before sufficient evidence mounts to include SSRIs as a standard in autism care [57], whereas SSRIs use in children, especially as early intervention, would certainly require a great amount of caution.

Ongoing investigations on SSRIs are nowadays focusing on issues of safety and efficacy in an attempt to assess the balance between possible gain and damage [49]. Pharmacogenomics research can tremendously contribute to this direction by identifying genetic predictors of individuals’ response and tolerability.

It has already been reported that 5-HTTLPR polymorphism may influence the individual responses to fluvoxamine administration in childhood autism [91]. In addition, animal studies have revealed that mice with functionally different allelic forms of *TPH2* and consequent impairment of serotonin synthesis do not respond to SSRIs, whereas the SSRIs antidepressant-like effect is reinstated, when the serotonin synthesis is enhanced by pre-treatment with tryptophan. It has been, conclusively, proven that administration of a serotonin precursor stimulates serotonin synthesis and, subsequently, improves SSRIs effect in mice carrying the mutated enzyme [16,19].

Therefore, in addition to SSRIs, other serotonergic agents, such as 5-HTP, could be probably considered as alternative, developmental interventions. Tryptophan depletion has already been reported to cause deterioration in patients with autism [64], whereas an oral challenge with 5-HTP had no impact in controls, but led to increase in serotonin blood concentration in patients [31]. Surprisingly, the clinical effects of 5-HTP administration in individuals with autism have not yet been studied.

5-HTP is commercially produced by extraction from the plant Griffonia simplicifolia and is typically available as the L-enantiomer, where most of the biological activity resides [95]. Its half-life is relatively short (4.3 ± 2.8 hours) [102] and the time to maximal concentration is 1–2 hours [62]. It is well absorbed from an oral dose with approximately 70% ending up in the bloodstream [60,61] and its absorption is not affected by the presence of other amino acids [11].

Contrary to L-tryptophan, 5-HTP crosses the blood-brain barrier with no requirement for a transport molecule. Its primary function is the elevation of serotonin within the CNS. Unlike L-tryptophan, 5-HTP cannot be shunted into niacin or protein production and is exclusively used on the metabolic pathway of serotonin synthesis. A number of studies have investigated the effect of 5-HTP in depression, reporting similar efficacy and tolerability to SSRIs. 5-HTP has, additionally, been used in fibromylagia, insomnia, obesity etc [11].

One major concern regarding the use of 5-HTP would be the risk of eosinophilia-myalgia syndrome. The disease presented in the 1980s, due to contamination of commercial tryptophan supplements. It was associated with faulty production methods using bacterial fermentation and, therefore, it would be rather unlikely to occur after use of the naturally produced 5-HTP [11,35,67]. Nevertheless, the administration of 5-HTP would require constant and close monitoring. A second concern is a potential serotonin syndrome, resulting from excess amounts of serotonin in the peripheral circulation. This can be easily prevented with the co-administration of 5-HTP and carbidopa. Carbidopa is a peripheral decarboxylase inhibitor, diminishing 5-HTP peripheral conversion into serotonin and, consequently, improving its availability in the CNS [95].

Taken into account that a number of neuropathological changes in the autistic brain are highly associated with the reduced CNS serotonin levels and that 5-HTP has an obvious role in increasing serotonin synthesis in the brain, it would be reasonable to consider the use of 5-HTP as an alternative, developmental intervention for children with autism.

However, the potential administration of 5-HTP as early treatment would arise a multitude of limitations. 5-HTP has not yet been widely tried in populations of adult patients with depression and has certainly not been tried in autism. Prior to its administration to children, a lot of questions regarding efficacy and safety should be addressed. Secondly, the reduced CNS serotonin might only represent a small part of a more generalized disruption, which causes the autistic phenotype and can obviously not be treated merely by serotonergic agents. Thirdly, any developmental serotonergic treatment would largely target the reduced serotonin levels in early life of patients. However, serotonin synthesis in autistic children is still being under investigation, whereas human studies only concern ages older than 2 years. Last but not least, it has not been examined whether early treatment with 5-HTP would have side effects over both the short and the long term [10]. Therefore, a large number of experiments on animals, as well as animal models for autism, would be utterly necessary before any early pharmacological intervention, especially in the form of serotonergic agents, is initiated in a human study.

## CONCLUSION

According to accumulating evidence from imaging, neuropathological and genetic studies, autism is a disorder of developmental arrest and, therefore, requires early treatment. Developmental intervention can utilize brain plasticity to provide permanent and substantial benefit. Up to now, early treatment consists solely of occupational or physical therapy, while no pharmacological intervention has been systematically used. Ongoing research aims to ascertain the safety and efficacy of various agents, including the ones targeting the disrupted serotonergic system. It has been speculated that part of the early damage could be prevented by providing the autistic brain with adequate serotonin levels. The use of SSRIs in autism is currently under intense research, whereas another alternative developmental intervention, the early administration of 5-HTP, is being suggested as a potentially promising option. However, any pharmacological early intervention needs to be largely tried on animals, as well as animal models for autism, before any human studies are instigated.

The identification of safe and efficacious pharmacological developmental intervention, along with related issues of pharmacogenomics, are essential parts of future research in autism.
